# A teenager with uncontrolled hypertension: a case report

**DOI:** 10.1186/1756-0500-5-659

**Published:** 2012-11-29

**Authors:** Abdul Wadud Chowdhury, ATM Hasibul Hasan, SME Jahan Kabir, KM Nurus Sabah

**Affiliations:** 1 Department of Cardiology, Dhaka Medical College Hospital, Dhaka, Bangladesh; 2 Department of Medicine (Outdoor Patient Department), Dhaka Medical College Hospital, Dhaka, Bangladesh

**Keywords:** Takayasu’s Arteritis (TA)

## Abstract

**Background:**

Takayasu Arteritis is a vasculitis occurring mostly in young females which may present in diverse ways. Here we report a teenager with Takayasu Arteritis who presented with uncontrolled hypertension. This case depicts an atypical presentation of this disease where the girl visited many physicians for controlling the level of hypertension and put a diagnostic dilemma about the underlying etiology of young hypertension.

**Case presentation:**

A 13 year old girl presented with epistaxis, persistent headache and uncontrolled hypertension. Her clinical examination revealed normal radial, very feeble femoral and absent other lower limb pulses. There was a blood pressure discrepancy of 50/40 mm of Hg between two arms. There were bruits over multiple areas including the abdominal aorta. She had features of left ventricular hypertrophy. Her Arch aortogram showed hugely dilated arch of aorta which became abruptly normal just after origin of left subclavian artery. There was ostio-proximal stenosis of right bracheocephalic artery, left common carotid and left subclavian artery with post stenotic dilatation of all the vessels. Abdominal aortogram revealed critical stenosis of abdominal aorta above the origin of renal arteries with a pressure gradient of 80/11 mm of Hg.

**Conclusion:**

Takayasu’s Arteritis should also be kept in mind while searching for the cause of uncontrolled hypertension in the young age group.

## Background

The estimated prevalence of hypertension in pediatric age group is between 2%–5% [1]. The usual form of hypertension in young is attributable to secondary causes. The most common cause is the renovascular one (60–70%) [2,3]. Cushing syndrome, hyperthyroidism, pheochromocytoma, essential hypertension, coarctation of aorta, SLE are also found less commonly with hypertension in children and adeloscent [4]. With the growing knowledge and awareness of hypertension, the rate of diagnosis is increasing in children [1]. Evidences are increasing regarding early development of atherosclerosis in child and their possible relation to hypertension and coronary artery disease [5]. Several studies have reported the correlation between pediatric hypertension and family H/O hypertension, low birth weight, excess body weight [6,7]. Here we describe a 13 year old girl presenting with epistaxis, headache and uncontrolled hypertension despite poly drug therapy, abnormal peripheral pulses and unequal blood pressure in upper limbs. Further investigations were done to determine the cause of hypertension. The arch and abdominal aortography further correlated the uncontrolled hypertension with Takayasu’s disease according to American College Rheumatology (ACR) criteria. KS Chugh et al. described Takayasu Arteritis as the most common cause of renovascular hypertension in India [8]. Takayasu Arteritis is a large vessel vasculitis of unknown origin characterized by granulomatous inflammation of aorta and its major branches, leading to stenosis, thrombosis and aneurysm formation.

## Case presentation

**Figure 1:**
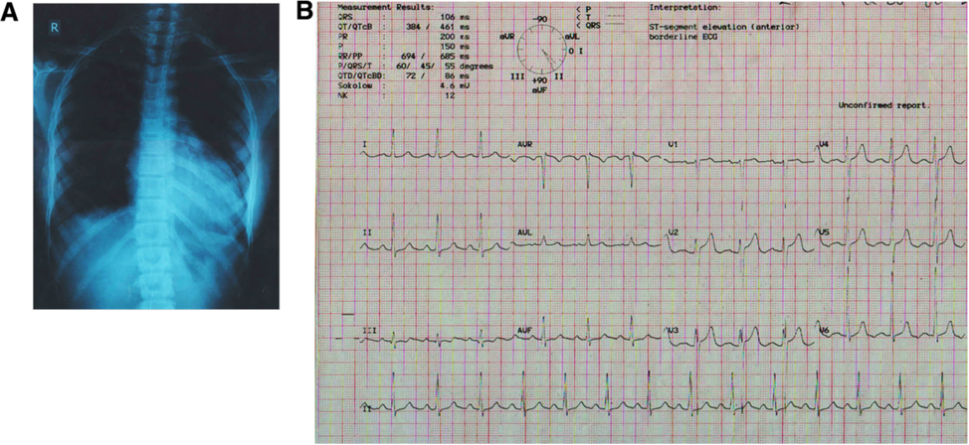
**A: CXR P-A view.** Cardiomegaly with LV type apex. **B** ECG. Left ventricular hypertrophy.

A 13 year old girl presented with three episodes of spontaneous profuse nasal bleeding within last three years which had remission without specific therapy. She had diffuse persistent headache without nausea or vomiting and uncontrolled hypertension, despite taking amlodipine and atenolol. She gave no H/O chest pain, shortness of breath, fever, prolonged cough, pulsatile tinnitus, light headedness, arthralgia, skin rash, weight loss, claudication or colour changes on cold exposure. There was no history of contact with TB patient. She did not give any H/O dizziness or syncope. On examination, both radial pulses were 80 beats/min, regular, high volume and surprisingly apparently symmetrical on both sides. There was no radio-femoral delay. Both the femoral pulses were feeble. All other lower limb pulses were absent. BP on right arm was 120/80 mmHg and on left arm was 170/120mmHg. There were bruits over both carotids, suprasternal, supraclavicular areas and over abdominal aorta. On precordial examination-apex beat was palpable at left 5th intercostal space just lateral to the midclavicular line. It was heaving in nature. A_2_ was loud, there was no added sound. All other systemic examinations including optic fundi were normal. On investigation, Hemoglobin was 11.2 gm/dl, Total Count-5100/mm [3], Neutrophil- 51%, Lymphocyte- 35%, Monocyte- 03%, Eosinophil-07%, Erythrocyte sedimentation rate (ESR)- 30 mm in 1st hour. Mantoux test (MT) and C-reactive protein (CRP) were negative. Blood glucose, Serum creatinine, urine analysis were normal. Chest X-ray showed cardiomegaly with LV type apex (Figure 1A).

**Figure 2:**
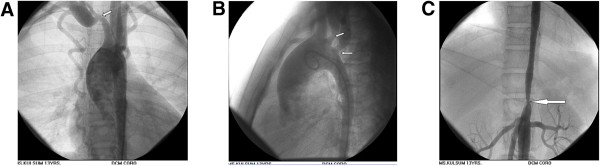
**A: Arch Aortogram.** Hugely dilated (70 mm) arch of aorta which became abruptly normal (35 mm) just after origin of left subclavian artery. Right bracheocephalic artery had ostio-proximal stenosis with marked post stenotic dilatation (white arrow). **B**: Arch Aortogram. Ostio-proximal stenosis of left common carotid and left subclavian artery with post stenotic dilatation (white arrow). **C**: Abdominal Aortogram critical stenosis of abdominal aorta (8.9 mm) above the origin of renal arteries (white arrow).

**Figure 3:**
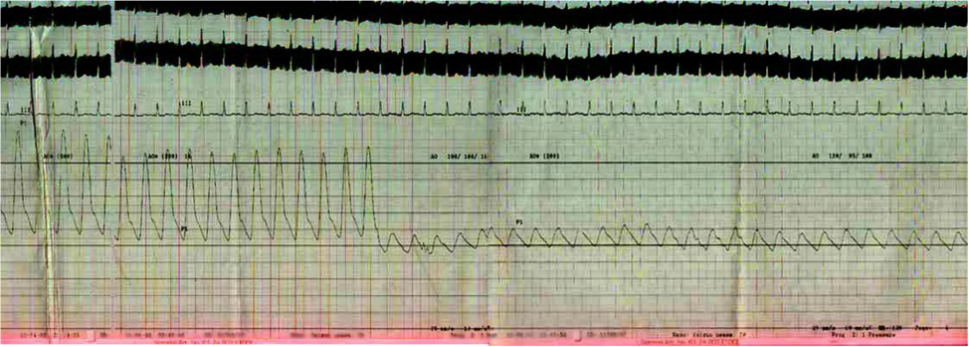
Pressure tracing in abdominal aorta.

ECG fulfilled the voltage criteria of left ventricular hypertrophy (Figure 1B). 2D, M-mode and Doppler echocardiography revealed concentric left ventricular hypertrophy, aneurysmal dilatation of aortic arch, proximal stenosis and post stenotic dilatation of brachiocephalic, left common carotid and left subclavian artery and narrowing of descending thoracic aorta beyond the origin of left subclavian artery. Arch Aortogram showed hugely dilated (70 mm) arch of aorta which became abruptly normal (35 mm) just after origin of left subclavian artery. Right bracheocephalic artery had ostio-proximal stenosis with marked post stenotic dilatation (Figure 2A: white arrow). There was also ostio-proximal stenosis of left common carotid and left subclavian artery with post stenotic dilatation (Figure 2B: white arrow). Abdominal aortogram revealed critical stenosis of abdominal aorta (8.9 mm) above the origin of renal arteries (Figure 2C: white arrow). Renal arteries were however normal. Pressure study in abdominal aorta showed a pressure tracing of 200/106 mm of Hg above and 120/95 mm of Hg below the stenosis (Figure 3).

She fulfilled four of the six major ACR (American College of Rheumatology) criteria for Takayasu’s disease eg, onset of age is 13 years (<40years), 50 mm of Hg pressure difference between systolic BP between arms (>10mm of Hg pressure difference), subclavian and aortic bruit and narrowing of major branches of aorta. She was prescribed Amlodipine 10 mg, Atenolol 100mg, Prazosin 6mg, Hydrochlorthiazide 50mg and Amiloride 5mg. Methotrexate 5mg weekly with Folinic acid supplementation were given too. Plan for Angioplasty and Stenting of abdominal aortic stenosis was provided to the patient.

Takayasu’s Arteritis (TA) is a chronic inflammatory large vessel vasculitis of unknown origin, predominantly affecting aorta and its major branches. It is also called Aortic arch syndrome, Pulse less disease, Occlusive thromboaortopathy, Martorell syndrome [9]. The first scientific description of Takayasu’s Arteritis was given by Mikito Takaysu, Professor of Opthalmology at Kanazawa University, Japan, in 1905 at 12th Annual conference of Japanese Ophthalmology Society [10]. He presented a 21year old woman with a peculiar form of arteriovenus anastomoses at optic fundi. K Onishi and T Kagosha also contributed with their patients of absent radial pulse in the same conference [10]. But the first ever documented description of this arteritis dates back to 1830. Rokushu Yamamoto who practiced Japanese oriental medicine, described a case of 45 years old man presenting with absent pulse in one upper limb and feeble pulse in another one following a year long history of high grade fever. During the period of follow up the patient subsequently became emaciated, dyspnoeic and died suddenly after 11 years [10]. The world wide prevalence of Takayasu’s disease is 3.3/million. The disease is more common in East Asia and in Asian descendants in other countries [11]. TA commonly presents in 2nd or 3rd decade of life, with a high female preponderance. But the female to male ratio declines from Eastern Asia to the West [12]. TA may manifest as asymptomatic pulseless disease to catastrophic neurological impairements. The disease may present in two phase, a prepulseless phase of nonspecific inflammatory signs, followed by a chronic phase of vascular insufficiency [13-15]. Presentation of TA varies among the races. Japanese patients are predominantly female, presents with pulslessness, dizziness, vertigo, aortic regurgitation, inflammatory process commonly affecting the arch and its major branches, whereas Indian patients are male dominant. Indian cases present with more hypertension, headache, LV hypertrophy and vasculitic involvement of abdominal aorta and renal arteries [15]. Diminished or absent pulse along with upper limb claudication and blood pressure difference is found in 84–96% of cases [16]. Vascular bruits involving carotid, subclavian and abdominal vessels are also common (80–94%) [17]. Hypertension is associated with 33–83% patients of TA [15,17]. Our index case was also a young girl with feeble femoral pulse and absent other peripheral pulses in lower limb, blood pressure discrepancy between arms, bruits over multiple areas of chest and neck and hypertension. The blood pressure discrepancy of 50/40 mm of Hg is probably due to the difference in percentage of stenosis among the brachiocephalic (70–80% stenosis) and left subclavian vessels (50–60% stenosis). Retinopathy, aortic regurgitation, congestive heart failure, cardiomyopathy, myocardial ischemia, headache, dizziness, seizure are less common association of TA. From common findings of TA, American College of Rheumatology has devised some diagnostic criteria for TA in 1990. Angiography remains the gold standard investigation for diagnosis. The main differential diagnosis include other causes of large vessel vasculitis eg inflammatory vasculitis (Syphilis, Tuberculosis, Behchets, SLE); development abnormalities (Coarctation of aorta, Marfans syndrome) and neurofibromatosis. TA has been classified on the basis of angiographic findings. The new classification was described at Takayasu Arteritis Conference in 1994 based on vessel involvement. Type-I involving branches from aortic arch, Type-IIa denoting ascending aorta, aortic arch and its branches, Type-IIb including Type-Ia plus descending thoracic aorta. Type-III means descending thoracic aorta, abdominal aorta and/ or renal arteries. Type-IV involves abdominal aorta and/ or renal arteries. Type-V is combined features of Type-IIb and Type-IV [15]. Ishikawa classified different clinical groups based on natural history and complications. He described Group-I as uncomplicated disease with or without pulmonary artery involvement, Group-IIA as mild/moderate single complication together with uncomplicated disease, Group-IIB as severe single complication together with uncomplicated disease, Group-III as two or more complications together with uncomplicated disease [17]. Ishikawa defined Takayasu retinopathy, Secondary hypertension, Aortic regurgitation, Aneurysm formation as four most important complications. Our index case met the angiographic criteria of Type-IV Takayasu Arteritis class and Group-III of Ishikawa class [17]. Ishikawa class caries a prognostic significance not only for the Japanese patients but also for the Indians. The overall five year survival rate is 83%. The survival rate is 100% in Group-I and 70% in Group-IIb and Group-III. The most common cause of mortality is cerebrovascular disease and cardiac failure. Regarding treatment strategy steroid had been the mainstay of treatment. Shelhamer et al. showed half of the TA patients on steroid won’t respond [18]. Kerr et al. showed overall remission rate of 33% with immunosuppressive drugs in steroid unresponsive patients [16]. Methotrexate though not more efficacious than others, became popular due to its well tolerability [19,20]. The combination of steroid and methotrexate demonstrated a remission rate of 81% in steroid unresponsive patients [21]. Treatment of hypertension and prevention of thrombosis are also important aspects of therapy. Treatment of hypertension with ACE inhibitors requires careful monitoring for renal artery stenosis. Surgery may be needed in patients with critical renal artery stenoses, limb claudication limiting the daily activities, stenosis of three or more cerebral vessels, moderate aortic regurgitation. Stenoses of renal artery are best treated by Percutaneous Transluminal Angioplasty [21]. Stent placement following angioplasty is a safe and effective procedure [22]. Takayasu’s Arteritis is a chronic progressive vasculopathy. So long term follow up is recommended. Markers of acute phase response are unreliable during follow up. Doppler studies and MRA are can help to determine the vessel wall thickness and lumen configuration.

## Conclusion

Takaysu’s Arteritis can have varied presentation. So a young female patient presenting with absent pulse, unequal blood pressure between arms and hypertension should be suspected clinically for Takayasu’s disease.

## Consent

Written informed consent was obtained from the patient’s guardian for publication of this case report and for all the accompanying images.

## Competing interests

The authors declare that they have no competing interests.

## Authors’ contributions

AWC is the first author and was involved in diagnosis by performing the angiography and writing a part of the manuscript. ATMHH is the communicating author and was involved in writing the manuscript. SMEJK and KMNS were responsible for the management of the patient. All the authors read and approved the final manuscript.
